# Anlotinib monotherapy in recurrent or metastatic nasopharyngeal carcinoma: a multicenter case-series analysis

**DOI:** 10.2478/raon-2025-0059

**Published:** 2025-12-16

**Authors:** Guan-Jie Qin, Yi-Xin Su, Yong Liang, Bin Zhang, Yu-Fei Pan, Jian-Xun Lu, Yue-Yun Xie, Jin-Xuan Dai, Ke-Quan Chen, Feng-Fei Qin, Hui-Yun Yang, Xiang-Yun Kong, Yuan Xie, Xiao-Lan Ruan, Yun-Yan Mo, Ru-Yun Zhang, Jian Zhang, Wei Jiang

**Affiliations:** 1 Department of Radiation Oncology, Affiliated Hospital of Guilin Medical University, Guilin, China; 2 Key Laboratory of Functional Genomics and Precision Prevention and Treatment of Oncology, Education Department of Guangxi Zhuang Autonomous Region, Guilin Medical University, Guilin, China; 3 Department of Radiation Oncology, Lingshan People’s Hospital, Lingshan, China; 4 Department of Oncology, Guiping People’s Hospital, Guiping, China; 5 Department of Radiation Oncology, Wuzhou Red Cross Hospital, Wuzhou, China; 6 Department of Oncology, Nanxishan Hospital of Guangxi Zhuang Autonomous Region, Guilin, China; 7 Department of Oncology, Nanning First People’s Hospital, Nanning, China; 8 Department of Oncology, The 924th Hospital of the Chinese people’s Liberation Army, Guilin, China; 9 Department of Oncology, Laibin People’s Hospital, Laibin, China

**Keywords:** anlotinib, nasopharyngeal carcinoma, recurrent, metastasis, tyrosine kinase inhibitors (TKI)

## Abstract

**Background:**

Anlotinib has shown encouraging therapeutic effect on various solid tumors. This study assessed the efficacy and safety of anlotinib monotherapy in patients with recurrent or metastatic nasopharyngeal carcinoma (rmNPC).

**Patients and methods:**

This study retrospectively included 30 patients with rmNPC, most following at least one previous line of systemic therapy. Patients underwent anlotinib monotherapy (12 or 10 mg/day). The primary endpoint was objective response rate (ORR). The secondary endpoints included progression-free survival (PFS), overall survival (OS), and toxicity.

**Results:**

Thirteen patients (43.3%) had metastatic NPC, 10 (33.3%) had recurrent NPC, and 7 (23.3%) had both meta-static and recurrent NPC. Twenty-two patients (73.3%) were platinum-refractory, and 23 (76.7%) received at least three cycles of anlotinib therapy. The best overall response was partial response observed in four patients, stable disease in 18, and progressive disease in eight. The ORR was 13.3% (95% CI, 0.4-26.2%) and disease control rate was 73.3% (95% CI, 56.5-90.1%). The median OS and PFS were 11.5 months (95% CI, 7.5-15.5) and 5.7 months (95% CI, 4.7-6.7), respectively. The relatively common grade 3 or higher adverse events were hand-foot syndrome (13.3%) and oral mucositis (13.3%).

**Conclusions:**

Anlotinib monotherapy demonstrated positive efficacy in patients with rmNPC. It was well tolerated by these patients and had acceptable toxicity.

## Introduction

Nasopharyngeal carcinoma (NPC) has a tremendous heterogeneity in geographical distribution and is highly prevalent in southern China. The age-standardized rates in prevalence areas are 50-100 times higher than in non endemic areas of the world.^1^ About 70% of patients present with locoregionally advanced disease at diagnosis, and approximately 30% of these eventually develop locoregional recurrence or distant metastasis, even after radical chemoradiotherapy.^2,3^ The combination of gemcitabine and cisplatin chemotherapy with or without PD-1 inhibitor-based immunotherapy is the first-line therapy for recurrent or metastatic nasopharyngeal carcinoma (rmNPC).^4–7^ No commonly accepted second-line therapy is available for platinum-refractory patients.^8^ Even with a growing number of treatment choices for patients with platinum-refractory rmNPC in recent clinical studies, only modest improvement in survival has been achieved, the median progression-free survival (PFS) being only 1.6–13.8 months.^9–11^

Angiogenesis induction has been considered as one of the ten hallmarks of cancer.^12^ Recently, the clinical application of tyrosine kinase inhibitors (TKI) targeting proangiogenic receptors, particularly the vascular endothelial growth factor receptor (VEGFR) family, has significantly improved the survival of several solid tumors, including NPC.^13–17^ Our previous study showed that apatinib, a novel small-molecule VEGFR signaling pathway inhibitor, achieved a high objective response rate (ORR) of 36.4% in patients with rmNPC who had experienced first-line treatment failure.^17^ However, apatinib tolerance in patients with rmNPC was poor, with 57.6% of the patients required dose adjustment during treatment.^17^

Anlotinib hydrochloride is a novel multi-target TKI used to treat angiogenesis and proliferative signaling of tumors, with high efficacy by inhibiting VEGFR-1-3, platelet-derived growth factor receptor-α and β, fibroblast growth factor receptor-1-4, c-Kit, and Ret.^18^ The phase 3 ALTER0303 trial showed that anlotinib monotherapy as third-line treatment of advanced non-small cell lung cancer can significantly prolong the median PFS from 1.4 months to 5.4 months and the median overall survival (OS) from 6.3 months to 9.6 months.^19^ Anlotinib showed moderate efficacy and tolerable toxicity in patients with platinum-refractory ovarian cancer.^20^ This data analysis was conducted in patients with rmNPC who had failed prior platinum-based chemotherapy or were unwilling to undergo further chemotherapy, aiming to investigate the clinical efficacy and safety of anlotinib in these patients.

## Patients and methods

### Patients of the study

This study retrospectively included patients with histopathologically diagnosed rmNPC from eight centers in the Guangxi Zhuang Autonomous Region, China, between January 1, 2019, and September 30, 2021. Patients were eligible for anlotinib treatment due to the following reasons: 1) no standard second-line treatment was available for rmNPC; 2) further chemotherapy was contraindicated; 3) refusal of chemotherapy due to toxicity from prior treatment. The inclusion criteria were: 1) histologically and/or radiographically confirmed rmNPC; 2) adequate hematological and biochemical parameters (absolute neutrophil count ≥ 1.5×10^9^/L; haemoglobin level ≥ 90 g/L; total bilirubin up to 1.5 times the upper limit of normal; alanine aminotransferase, aspartate aminotransferase or alkaline phosphatase up to 2.5 times the upper limit of normal; serum creatinine up to 1.5 times the upper limit of normal); 3) performance status of 0-2 (Eastern Cooperative Oncology Group [ECOG]); 4) measurable lesion according to RECIST Version 1.1 criteria; and 5) disease progression after platinum-based chemotherapy (evaluated by RECIST Version 1.1) or refusal to receive further chemotherapy. Exclusion criteria included: 1) prior treatment with antiangiogenic drugs before anlotinib therapy; 2) involvement or invasion of a major vascular structure by the tumor; 3) serious comorbidities that could potentially affect patient survival; 4) pregnant or breastfeeding women.

This study was approved by the ethics committees of all eight participating centers and adhered to the Declaration of Helsinki and Good Clinical Practice guidelines. All patients provided written informed consent before initiation of anlotinib treatment, and confidentiality of their medical data was maintained.

### Treatment

One cycle of oral anlotinib consisted of once-daily administration on days 1-14 followed by a pause of seven days.^19^ The initial anlotinib dose was 12 or 10 mg/day, based on the investigators’ assessment and the patients’ general conditions. Dose modification to 10 or 8 mg/day was permitted based on treatment-related toxicities. When ≥ Grade 2 adverse reactions occurred, anlotinib treatment would be interrupted until the adverse reaction recovered to < Grade 2. If tolerated, the patient continued with the same anlotinib dosage; otherwise, the dose was adjusted. Treatment was permanently discontinued if the patient experienced persistent toxicities despite dose adjustments or if progressive disease (PD) was documented.

### Patient evaluation and follow-up

Objective treatment response was assessed according to RECIST Version 1.1, based on imaging examinations performed at baseline and every two cycles of anlotinib treatment until treatment discontinuation or disease progression. Adverse events were recorded and graded according to the Common Terminology Criteria for Adverse Events (CTCAE, Version 5.0) during anlotinib treatment. Physical examination and laboratory parameters were recorded at baseline and at each follow-up examination during treatment. Laboratory tests and safety assessments were conducted within one month after the last dose of anlotinib in patients who discontinued treatment.

### Statistical analysis

The ORR was the primary endpoint, and secondary endpoints included disease control rate (DCR), PFS, OS, and adverse events. ORR was calculated as the proportion of patients with complete response (CR) and partial response (PR) in the entire cohort. DCR was calculated as the proportion of patients with CR, PR, and stable disease (SD) in the entire cohort. PFS was calculated from the start of anlotinib treatment to the time of PD or death from any cause. OS was defined as the time from the start of anlotinib treatment to death from any cause. Survival endpoints were censored at the time of the last follow-up for patients who reached the end of the study without progression or death.

The 95% confidence intervals (CI) of the ORR and DCR were calculated using the Clopper-Pearson method. Survival curves were estimated using Kaplan-Meier method. Multivariable analysis was performed using the Cox proportional hazards analysis and backward stepwise selection. Adverse events were summarized by frequency and percentage. Statistical significance was defined as a two-sided P-value < 0.05. All statistical analyses were performed using IBM SPSS Statistics for Windows, Version 24.0 (IBM Corp, Armonk, NY, USA).

## Results

### Patient characteristics

A total of 30 eligible patients with rmNPC were included. The median age of the participants was 55 years (range, 35-76 years). Table 1 summarizes the baseline characteristics of all included patients. Most patients (n = 28; 93.3%) were disease stage III or IV at diagnosis. Among the included patients, 10 (33.3%) had locoregional recurrence (nasopharynx, cervical lymph nodes, or both), 13 (43.3%) had distant metastatic disease (nine after chemoradiotherapy and four at the time of diagnosis), and 7 (23.3%) had both locoregional recurrence and distant metastasis. The most common organ involved in metastasis was the liver (11 patients; 36.7%). Anlotinib was administered after one line of chemotherapy to 13 (43.3%) patients, while 9 (30.0%) received at least two prior lines of systemic therapy before anlotinib treatment. Sixteen patients (53.3%) had platinum-refractory disease. Of the remaining 14 patients, eight rejected chemotherapy and used anlotinib as first-line treatment (two patients were older than 70 years; one patient had chronic renal insufficiency; five patients refused chemotherapy due to hematological toxicity from previous chemoradiotherapy), three could not continue chemotherapy due to toxicity from first-line platinum based chemotherapy, and three did not receive a platinum-containing regime as first-line treatment for personal reasons.

**TABLE 1.** Baseline characteristics of 30 patients with recurrent or metastatic nasopharyngeal carcinoma treated with anlotinib monotherapy

Characteristics	No. of patients (%)
Age (years)	
Median (55)	
Range (35–76)	
Sex	
Male	25 (83.3)
Female	5 (16.6)
ECOG status	
0-1	24 (80.0)
2	6 (20.0)
Disease stage^a^ at diagnosis	
Stage II	2 (6.7)
Stage III	9 (30.0)
Stage IV	19 (63.3)
Disease status when starting anlotinib therapy	
Local recurrence	10 (33.3)
Metastatic disease^b^	13 (43.3)
Both	7 (23.3)
Metastatic sites	
Liver	11 (36.7)
Lung	10 (33.3)
Bone	10 (33.3)
Distant lymph node	8 (26.7)
Number of prior systemic therapy lines	
0	8 (26.7)
1	13 (43.3)
≥ 2	9 (30.0)
Platinum-refractory disease^c^	
Yes	16 (53.3)
No	14 (46.7)
Anlotinib starting dose	
12 mg	21 (70.0)
10 mg	9 (30.0)
RT for recurrent/metastatic lesions	
Yes	3 (10.0)
No	27 (90.0)
Surgical treatment for recurrent lesions	
Yes	2 (6.7)
No	28 (93.3)
Immunotherapy before anlotinib	
Yes	3 (10.0)
No	27 (90.0)

a According to the 8^th^ American Joint Committee on Cancer Stage (AJCC) staging system.

b Includes patients with metastatic disease at diagnosis and those after radical treatment.

c Patients with disease progression within 6 months after first-line chemotherapy with a platinum-containing regimen.

1 ECOG = Eastern Cooperative Oncology Group; RT = radiotherapy

### Treatment administration

The end of follow-up in this study was December 28, 2021, at which time nine patients were still alive, and four continued with the anlotinib treatment. The median follow-up time was 18.3 months (range, 12.5–24.1 months). A total of 173 cycles of anlotinib were administered to the 30 patients (median, 4 cycles; range, 1-21 cycles), with 23 (76.7%) patients receiving more than three cycles and 6 (20.0%) receiving over ten cycles. The anlotinib starting dose was 12 mg/day in 21 patients (70.0%) and 10 mg/day in 9 (30.0%). Dose reductions were uncommon, occurring in only 4 (13.3%) patients (from 12 mg/day to 10 mg/day due to Grade 3 oral mucositis in two patients, and from 10 mg/day to 8 mg/day due to Grade 3 hand-foot syndrome [HFS] in one patient and Grade 2 hypothyroidism in another). Drug interruption occurred in 8 patients (26.7%) to alleviate adverse reactions (four for oral mucositis, two for HFS, and two for nasal bleeding). Overall, 10 (33.3%) patients had dose reduction or discontinuation of treatment due to toxicities (of these, two patients continued treatment with a reduced dose after treatment interruption). By the end of the study, 26 (86.7%) patients had discontinued therapy, 20 (66.7%) due to disease progression, and 6 (20.0%) for other reasons, including unacceptable toxicity related to anlotinib (n = 2), change to other treatment options (n = 2), voluntary termination of antitumor therapy (n = 1), and loss to follow-up for unknown reason (n = 1).

### Treatment efficacy

**FIGURE 1.**
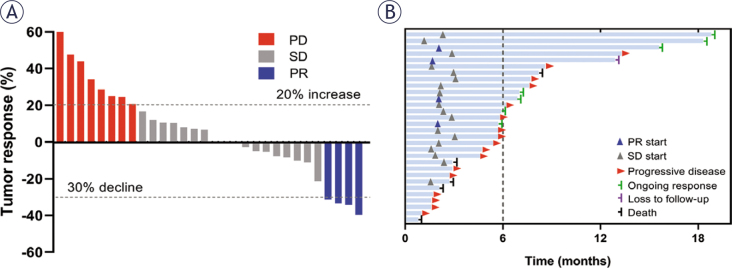
Treatment efficacy of anlotinib in patients with recurrent or metastatic nasopharyngeal carcinoma. **(A)** Waterfall plot shows the maximum change in target lesion diameter relative to baseline in each patient, following RECIST criteria, Version 1.1. **(B)** Swimmer’s plot shows the duration of response for each patient.

**FIGURE 2.**
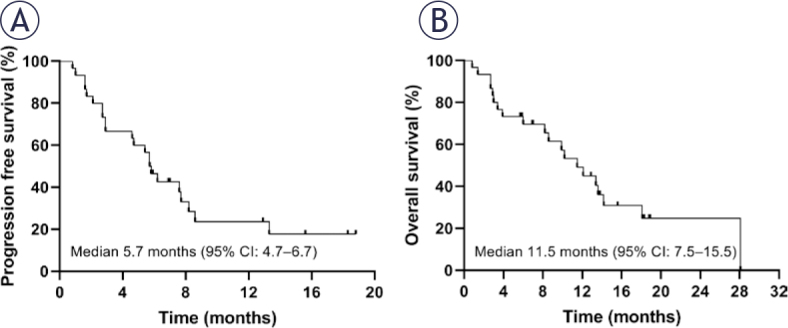
Survival curves for the 30 patients under anlotinib monotherapy in this study. **(A)** Progression-free survival and **(B)** overall survival.

Among the 30 evaluable patients, the best overall response was categorized as follow: PR in 4 patients (13.3%), SD in 18 patients (60.0%), and PD in 8 patients (26.7%). These results translated into an ORR of 13.3% (95% CI, 0.4–26.2%) and a DCR of 73.3% (95% CI, 56.5–90.1%; Figure 1A). Further analysis of the patients who achieved PR revealed that 2 patients maintained remission for more than three months, while the other 2 patients sustained remission for over six months (Figure 1B). By the cutoff date, a total of 22 patients (73.3%) experienced PD, with 20 of them succumbing to the disease (66.7%). In terms of survival outcomes, the median PFS was 5.7 months (95% CI, 4.7–6.7 months; Figure 2A), and the median OS was 11.5 months (95% CI, 7.5–15.5 months; Figure 2B).

### Toxicity

Toxicity data were collected and analyzed for the 30 patients who received anlotinib. The most common drug-related adverse events included HFS (n = 15; 50.0%), hypertension (n = 12; 40.0%), oral mucositis (n = 11; 36.7%), hypothyroidism (n = 9; 30.0%), and fatigue (n = 8; 26.7%) (Table 2). Bleeding events were observed in seven patients (23.3%), with four (13.3%) exhibiting Grade 1 urinary occult blood and three (10.0%) experiencing Grade 3 nasal bleeding. Other adverse events of ≥ Grade 3 severity included HFS (n = 4; 13.3%), oral mucositis (n = 4; 13.3%), hypertension (n = 1; 3.3%), and arthralgia (n = 1; 3.3%). All adverse events were deemed tolerable and manageable with appropriate interventions.

**TABLE 2.** Most common adverse reactions due to anlotinib treatment (incidence > 5%)

Adverse reaction	All grades	Grade 1	Grade 2	Grade 3	Grade 4
	*n* (%)				
Hand-foot syndrome	15 (50.0)	6 (20.0)	5 (16.7)	4 (13.3)	0
Hypertension	12 (40.0)	8 (26.7)	3 (10.0)	1 (3.3)	0
Oral mucositis	11 (36.7)	5 (16.7)	2 (6.7)	4 (13.3)	0
Hypothyroidism	9 (30.0)	7 (23.3)	2 (6.7)	0	0
Fatigue	8 (26.7)	5 (16.7)	3 (10.0)	0	0
Proteinuria	5 (16.7)	5 (16.7)	0	0	0
Positive urinary occult blood	4 (13.3)	4 (13.3)	0	0	0
Leukopenia	3 (10.0)	3 (10.0)	0	0	0
Nasal bleeding	3 (10.0)	0	0	3 (10.0)	0
Arthralgia	2 (6.7)	0	1 (3.3)	1 (3.3)	0

## Discussion

This study offers valuable insights into the efficacy and safety of anlotinib monotherapy in patients with rmNPC, the majority of whom had received at least one prior systemic treatment. The cohort demonstrated an ORR of 13.3% (95% CI, 0.4–26.2%), a DCR of 73.3% (95% CI, 56.5–90.1%). The median PFS was 5.7 months (95% CI, 4.7–6.7 months), and median OS was 11.5 months (95% CI, 7.5–15.5 months). Grade 3 and 4 toxicities were relatively infrequent, with 10 patients (33.3%) undergoing dose reductions or discontinuation of treatment due to adverse events. Overall, these findings indicate that anlotinib may offer efficacy and a tolerable safety profile in patients with rmNPC.

Patients with rmNPC typically experience disease progression within 7.0 months following chemotherapy.^4^ Recent phase III clinical trials have demonstrated that the combination of cisplatin, gemcitabine, and PD-1 monoclonal antibody can significantly extend the median PFS to 9.7–11.7 months. However, most patients eventually developed disease progression, resulting in a 1-year PFS of 45.8–49.4%.^5,6^ There is currently no standard salvage treatment for patients whose platinum-containing regimen. Previous studies have shown that single-drug chemotherapy or PD-1 monoclonal antibody monotherapy yields an ORR of 2.9–48%, a median OS was 7.6–17.1 months and a median PFS was 2.4–9.9 months.^9,13^ Our results indicate that anlotinib has comparable efficacy to these treatments, with a median PFS approaching six months.

The lack of a standardized treatment for platinum-refractory patients has prompted several clinical trials evaluating VEGF-VEGFR signaling pathway inhibitors in heavily pretreated patients. These studies have reported variable ORRs of 2.7–36.4% and median PFS of 1.8–5.0 months.^21–25^ For instance, sunitinib^21^ achieved an ORR of 7.1% and pazopanib^22^ achieved an ORR of 6.1%, while sorafenib^23^ demonstrated an ORR of 3.7%. Apatinib, which showed an ORR of over 30%, required dose modifications in over 40% of patients due to intolerable toxicities.^24^ In comparison, anlotinib exhibited a modest ORR and had relatively acceptable toxicities in this study. This result is consistent with another prospective phase II study, in which anlotinib had an ORR of 20.5% and a median PFS of 5.7 months in 39 patients with rmNPC treated with third line therapy.^25^

The most common adverse reactions in this study were consistent with those reported for targeted therapies in solid tumors. Grade 3 or higher adverse events included HFS (n = 4; 13.3%), oral mucositis (n = 4; 13.3%), nasal bleeding (n = 3; 10.0%), hypertension (n = 1; 3.3%), and arthralgia (n = 1; 3.3%). The relatively low toxicity of anlotinib may be attributed to its low half-maximal inhibitory concentration (IC_50_).^26^ Previous phase III clinical trials have reported that the incidence of Grade 3 oral mucositis associated with anlotinib treatment was as low as 1%.^19^ However, in this study, Grade 3 oral mucositis occurred at a higher rate and emerged as the primary reason for dose reduction or discontinuation of anlotinib. Similar findings were observed in a recent prospective phase II study, which demonstrated that the incidence of grade 3 oral mucositis during anlotinib treatment for rmNPC was 21.1% (8 of 38 patients), with 6 patients requiring dose adjustment.^25^ These results can be attributed to the widespread late radiation toxicity in patients with NPC who previously received high-dose radiotherapy, predisposing them to oral mucositis when using antiangiogenic drugs.

One of the risks associated with the use of tyrosine kinase inhibitors is bleeding, particularly in patients with recurrent NPC who have undergone radiotherapy and chemotherapy.^16,21^ Both sunitinib and pazopanib have been linked to fatal hemorrhagic events in patients with rmNPC.^21,22^ Phase II clinical studies reported a high bleeding risk for patients with rmNPC under sunitinib therapy, with 9 (64%) of the patients experiencing such events and 2 patients suffering from fatal bleeding.^21^ In a prospective study of 33 patients with rm-NPC treated with pazopanib, one patient experienced fatal bleeding.^22^ Adverse bleeding reactions were rarely reported in previous studies on the use of anlotinib in solid tumors.^27,28^ A recent prospective study did not identify any grade 3 bleeding events caused by anlotinib in patients with rmN-PC.^25^ In our study, Grade 3 nasal bleeding caused by anlotinib occurred in three patients (10%), two of whom had nasopharynx recurrence and one had liver and lung metastasis. No fatal hemorrhages or bleeding from other organs occurred in our study. Vascular radiation injury and the resulting regression or necrosis of tumor-invading vessels have been suggested as potential main cause of these bleeding events.^21^ Given the finding from previous studies and this study, the occurrence of bleeding events should be closely monitored during the clinical application of antiangiogenesis therapy in patients with rmNPC who have previously received radiotherapy, particularly in those with nasopharynx recurrence.

The current study has several limitations that should be acknowledged. First, the relatively small sample size and the short duration of follow-up may have introduced bias into the survival analysis, potentially obscuring the true efficacy of anlotinib. Second, the heterogeneous nature of the patient population, particularly the varying proportions of patients with liver metastasis, could have influenced the observed outcomes. Third, we were unable to investigate potential biomarkers associated with anlotinib efficacy, such as VEGFR-2 expression. Future studies should focus on exploring these biomarkers to better guide individualized treatment strategies.

In conclusion, this study provides evidence supporting the efficacy of anlotinib monotherapy in patients with recurrent or metastatic nasopharyngeal carcinoma (rmNPC). The treatment was generally well-tolerated, with a manageable toxicity profile. However, these findings require further validation through larger-scale, prospective studies.

## References

[1] Bray F, Ferlay J, Soerjomataram I, Siegel RL, Torre LA, Jemal A. (2018). Global cancer statistics 2018: GLOBOCAN estimates of incidence and mortality worldwide for 36 cancers in 185 countries. CA Cancer J Clin.

[2] Ren Y, Qiu H, Yuan Y, Ye J, Tian Y, Wen B (2017). Evaluation of 7th edition of AJCC staging system for nasopharyngeal carcinoma. J Cancer.

[3] Zhang MX, Li J, Shen GP, Zou X, Xu JJ, Jiang R (2015). Intensity-modulated radiotherapy prolongs the survival of patients with nasopharyngeal carcinoma compared with conventional two-dimensional radiotherapy: a 10-year experience with a large cohort and long follow-up. Eur J Cancer.

[4] Zhang L, Huang Y, Hong S, Yang Y, Yu G, Jia J (2016). Gemcitabine plus cisplatin versus fluorouracil plus cisplatin in recurrent or metastatic nasopharyngeal carcinoma: a multicentre, randomised, open-label, phase 3 trial. Lancet.

[5] Yang Y, Qu S, Li J, Hu C, Xu M, Li W (2021). Camrelizumab versus placebo in combination with gemcitabine and cisplatin as first-line treatment for recurrent or metastatic nasopharyngeal carcinoma (CAPTAIN-1st): a multicentre, randomised, double-blind, phase 3 trial. Lancet Oncol.

[6] Mai HQ, Chen QY, Chen D, Hu C, Yang K, Wen J (2021). Toripalimab or placebo plus chemotherapy as first-line treatment in advanced nasopharyngeal carcinoma: a multicenter randomized phase 3 trial. Nat Med.

[7] Yang Y, Pan J, Wang H, Zhao Y, Qu S, Chen N (2023). Tislelizumab plus chemotherapy as first-line treatment for recurrent or metastatic nasopharyngeal cancer: a multicenter phase 3 trial (RATIONALE-309). Cancer Cell.

[8] Wong KCW, Hui EP, Lo KW, Lam WKJ, Johnson D, Li L (2021). Nasopharyngeal carcinoma: an evolving paradigm. Nat Rev Clin Oncol.

[9] Lv JW, Li JY, Luo LN, Wang ZX, Chen YP. (2019). Comparative safety and efficacy of anti-PD-1 monotherapy, chemotherapy alone, and their combination therapy in advanced nasopharyngeal carcinoma: findings from recent advances in landmark trials. J Immunother Cancer.

[10] Chong WQ, Low JL, Tay JK, Le TBU, Goh GS, Sooi K (2025). Pembrolizumab with or without bevacizumab in platinum-resistant recurrent or metastatic nasopharyngeal carcinoma: a randomised, open-label, phase 2 trial. Lancet Oncol.

[11] Chan ATC, Lee VHF, Hong RL, Ahn MJ, Chong WQ, Kim SB (2023). Pembrolizumab monotherapy versus chemotherapy in platinum-pretreated, recurrent or metastatic nasopharyngeal cancer (KEYNOTE-122): an open-label, randomized, phase III trial. Ann Oncol.

[12] Hanahan D, Weinberg RA. (2011). Hallmarks of cancer: the next generation. Cell.

[13] Lee V, Kwong D, Leung TW, Lam KO, Tong CC, Lee A. (2017). Palliative systemic therapy for recurrent or metastatic nasopharyngeal carcinoma - How far have we achieved?. Crit Rev Oncol Hematol.

[14] Qin S, Li A, Yi M, Yu S, Zhang M, Wu K. (2019). Recent advances on anti-angiogenesis receptor tyrosine kinase inhibitors in cancer therapy. J Hematol Oncol.

[15] Lee NY, Zhang Q, Pfister DG, Kim J, Garden AS, Mechalakos J (2012). Addition of bevacizumab to standard chemoradiation for locoregionally advanced nasopharyngeal carcinoma (RTOG 0615): a phase 2 multi-institutional trial. Lancet Oncol.

[16] Hui EP, Ma BBY, Loong HHF, Mo F, Li L, King AD (2018). Efficacy, safety, and pharmacokinetics of axitinib in nasopharyngeal carcinoma: a preclinical and phase II correlative study. Clin Cancer Res.

[17] Ruan X, Liang JH, Pan Y, Cai R, Zhang RJ, He Z (2021). Apatinib for the treatment of metastatic or locoregionally recurrent nasopharyngeal carcinoma after failure of chemotherapy: a multicenter, single-arm, prospective phase 2 study. Cancer.

[18] Sun Y, Niu W, Du F, Du C, Li S, Wang J (2016). Safety, pharmacokinetics, and antitumor properties of anlotinib, an oral multi-target tyrosine kinase inhibitor, in patients with advanced refractory solid tumors. J Hematol Oncol.

[19] Han B, Li K, Wang Q, Zhang L, Shi J, Wang Z (2018). Effect of anlotinib as a third-line or further treatment on overall survival of patients with advanced non-small cell lung cancer: the ALTER 0303 phase 3 randomized clinical trial. JAMA Oncol.

[20] Cui Q, Hu Y, Ma D, Liu H. (2021). A retrospective observational study of anlotinib in patients with platinum-resistant or platinum-refractory epithelial ovarian cancer. Drug Des Devel Ther.

[21] Hui EP, Ma BBY, King AD, Mo F, Chan SL, Kam MKM (2011). Hemorrhagic complications in a phase II study of sunitinib in patients of nasopharyngeal carcinoma who has previously received high-dose radiation. Ann Oncol.

[22] Lim WT, Ng QS, Ivy P, Leong SS, Singh O, Chowbay B (2011). A Phase II study of pazopanib in Asian patients with recurrent/metastatic nasopharyngeal carcinoma. Clin Cancer Res.

[23] Elser C, Siu LL, Winquist E, Agulnik M, Pond GR, Chin SF (2007). Phase II trial of sorafenib in patients with recurrent or metastatic squamous cell carcinoma of the head and neck or nasopharyngeal carcinoma. J Clin Oncol.

[24] Huang L, Zhang X, Bai Y, Chua KLM, Xie Y, Shu X (2021). Efficacy and safety of apatinib in recurrent/metastatic nasopharyngeal carcinoma: a pilot study. Oral Oncol.

[25] Fang Y, Su N, Zou Q, Cao Y, Xia Y, Tang L (2023). Anlotinib as a third-line or further treatment for recurrent or metastatic nasopharyngeal carcinoma: a single-arm, phase 2 clinical trial. BMC Med.

[26] Lin B, Song X, Yang D, Bai D, Yao Y, Lu N. (2018). Anlotinib inhibits angiogenesis via suppressing the activation of VEGFR2, PDGFRβ and FGFR1. Gene.

[27] Shen G, Zheng F, Ren D, Du F, Dong Q, Wang Z (2018). Anlotinib: a novel multitargeting tyrosine kinase inhibitor in clinical development. J Hematol Oncol.

[28] Gao Y, Liu P, Shi R. (2020). Anlotinib as a molecular targeted therapy for tumors. Oncol Lett.

